# Temporal Dynamics of T Helper Populations in the Proximal Small Intestine after Oral Bovine Lactoferrin Administration in BALB/c Mice

**DOI:** 10.3390/nu13082852

**Published:** 2021-08-19

**Authors:** Mario Ynga-Durand, Gabriela Tapia-Pastrana, Xóchitl Abril Rebollar-Ruíz, Mariazell Yépez-Ortega, Oscar Nieto-Yañez, Ivonne Maciel Arciniega-Martínez, Aldo Arturo Reséndiz-Albor

**Affiliations:** 1 Laboratorio de Inmunidad de Mucosas, Sección de Estudios de Posgrado e Investigación, Escuela Superior de Medicina del Instituto Politécnico Nacional, Salvador Díaz Mirón y Plan de San Luis S/N, Miguel Hidalgo, Casco de Santo Tomas, México City 11340, Mexico; myngad1500@alumno.ipn.mx (M.Y.-D.); xrebollarr1800@alumno.ipn.mx (X.A.R.-R.); myepezo1200@alumno.ipn.mx (M.Y.-O.); 2 Laboratorio de Investigación Biomédica del Hospital Regional de Alta Especialidad de Oaxaca, San Bartolo Coyotepec 71256, Mexico; gtapia@hraeoaxaca.gob.mx; 3 Carrera de Médico Cirujano, Facultad de Estudios Superiores Iztacala, Universidad Nacional Autónoma de México, Tlalnepantla 54090, Mexico; o.nieto@comunidad.unam.mx

**Keywords:** IgA, mucosal immune system, intestinal modulation, TGF-β, Peyer’s patches, lamina propria

## Abstract

Bovine lactoferrin (bLf), a component of milk and a dietary supplement, modulates intestinal immunity at effector and inductor sites. Considering the regional difference in intestinal compartments and the dynamics of local cytokine-producing cells in the gut across time, the aim of this work was to characterize the effects of bLf on the proximal small intestine in a BALB/c murine model of oral administration. Male BALB/c mice were treated with oral bLf vs. saline control as mock by buccal deposition for 28 days. Intestinal secretions were obtained at different time points and cells were isolated from Peyer’s patches (PP) and lamina propria (LP) of the proximal small intestine as representative inductor and effector sites, respectively. Total and specific anti-bLF IgA and IgM were determined by enzyme-immuno assay; the percentages of IgA^+^ and IgM^+^ plasma cells (PC) and cytokine-producing CD4^+^ T cells of PP and LP were analyzed by flow cytometry. We found that total and bLf-specific IgA and IgM levels were increased in the intestinal secretions of the bLf group in comparison to mock group and day 0. LP IgA^+^ PC and IgM^+^ PC presented an initial elevation on day 7 and day 21, respectively, followed by a decrease on day 28 in comparison to mock. Higher percentages of CD4^+^ T cells in LP were found in the bLf group. Cytokines-producing CD4^+^ T cells populations presented a pattern of increases and decreases in the bLf group in both LP and PP. Transforming growth factor beta (TGF-β)^+^ CD4^+^ T cells showed higher percentages after bLf administration with a marked peak at day 21 in both LP and PP in comparison to mock-treated mice. Oral bLf exhibits complex immune properties in the proximal small intestine, where temporal monitoring of the inductor and effector compartments reveals patterns of rises and falls of different cell populations. Exceptionally, TGF-β^+^ CD4^+^ T cells show consistent higher numbers after bLf intervention across time. Our work suggests that isolated measurements do not show the complete picture of the modulatory effects of oral bLf in immunological sites as dynamic as the proximal small intestine.

## 1. Introduction

Bovine lactoferrin (bLf) is an iron-binding glycoprotein from the transferrin family that is secreted in cow’s milk and it is highly conserved across mammalian species [1]. Besides its well-known chemical properties involved in inflammation and defense against microorganisms, bLf has modulatory effects on the innate and adaptive immune response [2]. Specific receptors that mediate bLf interaction with immune cells have been identified and populations affected by its administration include antigen presenting cells as well as B and T lymphocytes [3]. This interplay results in a prominent influence on the balance of Th1 and Th2 responses, cytokine microenvironment and humoral responses [4,5]. Importantly, the immunomodulatory effects of bLf depend on its administration route [6] and oral treatment in small animals has resulted in upregulation of total and specific intestinal IgA, IgA^+^ B cells, and modulation of Th1 and Th2-related cytokine producing lymphocytes in the gut [6,7,8,9,10,11,12,13,14]. Importantly, analyses of the mucosal immune responses have revealed that temporal and regional dynamics are present in the gut [15]. Both inductor and effector compartments present differences between small and large intestine, and an immune sub-regionalization has been identified in the former that distinguishes proximal and distal sections [16]. bLf administration in mice has been shown to have dissimilar effects on each zone, with a higher increase in IgA and Th2 associated cytokines in the distal section in comparison to the proximal small intestine [17]. The human duodenum and jejunum (equivalent to the murine proximal section of the small intestine) is responsible for most nutrient absorption [18] and associated with celiac disease (an antigen-mediated chronic inflammatory disease) [19] as well as the induction of mucosal responses [20]. Notably, in addition to the dietary components, the proximal small intestine receives bile, a nutrient adsorption facilitator with important immunomodulatory activities, providing this section a particular immunological relevance. Information regarding the immune effect of bLf as a dietary supplement on this region is particularly relevant in light of its proposed long-term clinical use. The aim of this study was to analyze the effects of orally administered bLf on the IgA response and associated parameters in the classically defined inductive (Peyer patches) and effector compartments (lamina propria) of the proximal small intestine in healthy BALB/c mice [21], focusing on the temporal dynamics of the cellular compartment. Our results provide new evidence of the influence of oral bLf on IgA^+^ and IgM^+^ plasma cells (PC), total B cells, CD4^+^ and CD8^+^ T lymphocytes and cytokine producing population in the inductive and effector sites of the proximal small intestine.

## 2. Materials and Methods

### 2.1. Mice

Eight-week-old male BALB/c mice (25–30 g) were obtained from our Animal Breeding Unit (Escuela Superior de Medicina, IPN) and were housed in plastic cages in groups of three. All mice were kept on a 12 h light:dark cycle (lights on at 6 a.m.) for two weeks prior to their experimental manipulation at 10 weeks of age. Animals were handled according to a protocol (ESM-CICUAL-01/08-11-2019) in accordance with the Mexican federal regulations for animal experimentation and care (NOM-062-ZOO-1999, Ministry of Agriculture, Mexico City, Mexico) and the experiments were approved by the Institutional Animal Care and Use Committee of the Escuela Superior de Medicina, Instituto Politécnico Nacional.

### 2.2. Animal Diet

Food in oval pellet form and autoclaved purified water were provided ad libitum and exchanged every 24 h. The diet provided was 5001 Laboratory Rodent Diet (LabDiet, St. Louis, MO, USA).

### 2.3. Bovine Lactoferrin

Bovine lactoferrin (3% iron saturated) was purchased from NutriScience Innovations (Trumbull, CT, USA). Purity was confirmed by sodium dodecyl sulfate-polyacrylamide gel electrophoresis (SDS-PAGE) with Coomassie brilliant blue (Sigma, St. Louis, MO, USA) and silver staining. A solution of bLf containing 5 mg per 100 µL of vehicle (sterile saline solution 0.85% *w*/*v* NaCl) was prepared for the experimental interventions. We used a 5000 µg dose of bLf per day (approximately 200 mg/kg) as this was demonstrated have immunomodulatory effect in the murine gut [8,17]. bLf or mock treatment was administered in the morning (9–10 a.m.).

### 2.4. Experimental Design

Four groups of five mice were treated daily by buccal deposition with a micropipette containing 100 µL of sterile saline solution with 5 mg of bLf for 7, 14, 21 and 28 days. The control (mock group) consisted of mice receiving 100 µL of sterile saline solution for 7, 14, 21 and 28 days.

### 2.5. Collection of Samples

All mice were euthanized by an intraperitoneal injection of a lethal dose of 100 mg/kg body weight pentobarbital sodium salt (P3761, Sigma-Aldrich) and exsanguinated by cardiac puncture. The small intestine was dissected and the proximal segment (defined as the 5 cm segment following the pylorus) was flushed with 5 mL of sterile phosphate-buffered saline (PBS). Intestinal washings were centrifuged at 5000 rpm for 15 min at 4 °C and the supernatants were mixed with a protease inhibitor cocktail (Complete Mini, Roche Diagnostics, Mannheim, Germany) and stored at −70 °C until evaluation of total and specific antibodies.

### 2.6. Immunoglobulin Measurement

Determination of total IgA and IgM antibodies, and bLf-specific IgA in intestinal fluids was carried out by a modified standard ELISA as described previously [9]. Absorbance was measured at 490 nm by using an enzyme immunoassay reader (Sigma). To quantify total antibodies with standard curves, 100 µL of purified mouse IgM, or 100 µL of a serial dilution with a known concentration of mouse myeloma IgA was included in duplicates.

### 2.7. Peyer Patches and Lamina Propria Lymphocytes Isolation

The isolation of cells from Peyer’s patches and lamina propria from the small intestine was conducted as described previously [8,22].

### 2.8. Flow Cytometry Analysis

Thereafter, the cell suspensions were adjusted to 1 × 10^6^ cells/mL in PBS for cytofluorometric analysis of the anti-CD19 and anti-CD138 antibodies. For intracellular detection, the cells were fixed, permeabilized and stained with anti-IgA and anti-IgM. The surface phenotype of the T cells was detected using anti-CD3, anti-CD4, and anti-CD8a. Gating strategy and a representative plot are shown in Figure S1. To detect the intracellular signals of IL-4, IL-5, IL-6, IL-10, TGF-β, IFN-γ and TNF-α in CD4^+^ T cells, these analyses were conducted based on previously described protocols (Arciniega-Martinez et al. [8]). Gating strategy and a representative plot are shown in Figure S2. The fluorescence signal intensity was recorded and analyzed by a FACSCalibur flow cytometer (Becton Dickinson Biosciences San Jose, CA, USA). Events were collected from the lymphocyte gate on the FSC/SSC dot plot. A total of 20,000 gated events were acquired from each sample using BD FACSDiva^TM^ software 6.1 (Becton Dickinson Biosciences).

### 2.9. Statistical Analysis

Data are expressed as the mean ± standard deviation (SD) of five mice per group from three independent experiments. Multiple comparisons of immunological data between data points across time versus day 0 were analyzed by one-way ANOVA. If a significant main effect was identified (*p* ≤ 0.05), the means of the respective groups were compared using the post hoc Dunnett’s Multiple Comparison Test. Multiple comparisons of immunological data between the bLf intervention group versus mock were analyzed by two-way ANOVA with Bonferroni multiple comparisons correction. Significance was defined as a *p* ≤ 0.05. Non-linear regression (second order polynomial) was used to fit a curve for selected data. All analyses were performed using the program GraphPad Prism Version 6 software (GraphPad Software Incorporated, San Diego CA, USA).

## 3. Results

### 3.1. Oral bLf Increases the Total and Specific IgA and IgM Levels and Modulates the IgA^+^ and IgM^+^ PC Populations in the Proximal Small Intestine

**Figure 1:**
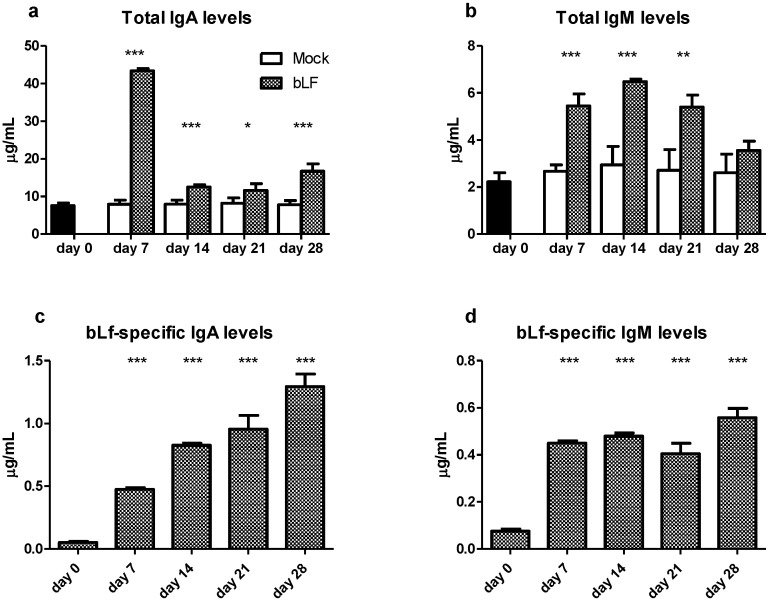

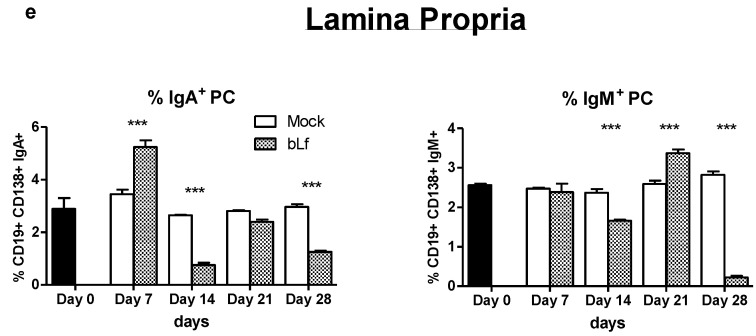
Effects of oral bLf vs. mock on the levels of total IgM (**a**) and IgM (**b**), and bLf-specific IgA (**c**) and IgM (**d**) in the intestinal secretions of the proximal small intestine. Mean and standard deviation (SD) are shown. Effects of oral bLf on the percentages of IgA^+^ and IgM^+^ plasma cells (PC)in lamina propria (LP) (**e**) of the proximal small intestine. Mean and SD are shown. Significant differences: * *p* ≤ 0.05, ** *p* ≤ 0.01, *** *p* ≤ 0.001, compared to mock in (**a**,**b** and **e**). Significant difference to day 0 is shown in 1(**c**,**d**. Non-significant results are omitted.

In order to study the antigen-specific and unspecific stimulation of bLf in the proximal small gut during oral administration, antibody response was measured in the intestinal secretions recollected from washing this dissected section. The analysis of antibody production at the proximal small intestine showed that mice treated with oral bLf present higher levels of total IgA and IgM at all time points after intervention compared to mock group (*p* ≤ 0.001 for all) except for IgM at day 28 (Figure 1a,b). Regarding the specific antibody response, the bLf-treated group showed higher levels of IgA and IgM at days 7, 14, 21 and 28 compared with day 0 (*p* ≤ 0.001 for all) (Figure 1c,d).

Due to the dynamic pattern of total IgA and IgM production in the proximal small gut, we decided to investigate the effect of oral bLf on the PC populations, as the main contributor to antibody production [23]. Plasma cell percentages (%) in the proximal small gut were analyzed. In the effector compartment (LP) of bLf-treated mice, a decreased percentage of IgA^+^ PC in comparison to mock was found at all time points after intervention except a transitory increase at day 7 (*p* ≤ 0.001). LP IgM^+^ PC percentages in the bLf group were lower than the mock group at days 14 and 28 (*p* ≤ 0.001), after a transitory peak in day 21 (*p* ≤ 0.001) (Figure 1e).

This combined result shows that oral bLf administration results in a specific humoral response in the proximal small gut that continuously increases over time, while non-antigen specific antibody response shows an initial peak of IgA and IgM secretion followed by a reduction that in the case of IgM returns to mock treated levels. These datasets reveal a complex modulating effect of oral bLf in the proximal small gut.

### 3.2. Oral bLf Modulates the Percentage of T Populations in the Effector and Inductor Compartments of the Proximal Small Intestine

**Figure 2:**
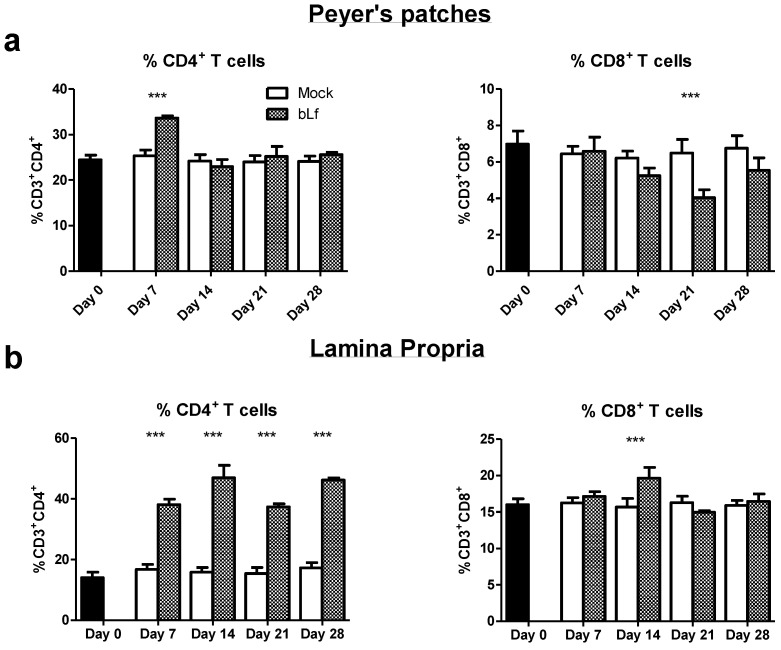
Effects of oral bLf vs. mock on the percentages of CD4^+^ and CD8^+^ T cells in PP (**a**) and LP (**b**) of the proximal small intestine. Mean and SD are shown. Significant differences: *** *p* ≤ 0.001 compared to mock. Non-significant results are omitted.

CD4^+^ T cells have been recognized as key drivers in humoral and cellular immunity. We investigated the effect of oral bLf on this subpopulation in the proximal small intestine. Mice treated with oral bLf had a higher percentage of CD3^+^CD4^+^ T cells at day 7 (*p* ≤ 0.001) and a lower percentage of CD3^+^CD8^+^ T cells at day 21 in the PP (*p* ≤ 0.001) (Figure 2a) in comparison to mock.

In LP, the bLf group showed higher percentages of CD3^+^CD4^+^ T cells in all days after intervention in comparison to mock (*p* ≤ 0.001). A transitory increase of CD3^+^CD8^+^ T cells percentages was found at day 14 (*p* ≤ 0.001) followed by no differences to mock in the rest of the analyzed time points (Figure 2b). These results show a marked and persistent increase of CD4^+^ T cells in the effector compartment during oral bLf treatment.

### 3.3. Oral bLf Modulates the Cytokine-Producing CD4^+^ T Cell Populations in the Effector and Inductor Compartments of the Proximal Small Intestine

**Figure 3:**
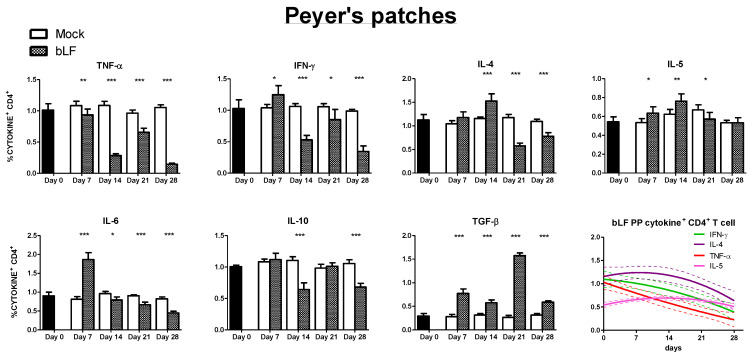
Effects of oral bLf vs. mock on the percentages of cytokine^+^ CD4^+^ T cells in PP of the proximal small intestine. Mean and SD are shown. Non-linear regression models were plotted for TNF-α, IFN-γ, IL-4 and IL-5. Significant differences: * *p* ≤ 0.05, ** *p* ≤ 0.01, *** *p* ≤ 0.001 compared to mock. Non-significant results are omitted.

CD4^+^CD3^+^ T cells mediate their effect in part through the different cytokines they secrete, and a subset classification associated to this production has been described [24]. Hence, we studied the effect of oral bLf on the cytokine production of this population in the proximal small gut. The following results were found in the PP (Figure 3): both Th1 associated cytokines, tumor necrosis factor alpha (TNF-α)^+^ and interferon gamma (IFN-γ)^+^ expressing CD4^+^ T cells percentage were lower than mock at every timepoint after intervention, except for a transitory peak of IFN-γ secreting cells at day 7 (*p* ≤ 0.05). With regard to Th2 associated cytokines in PP, IL-4 cells had a peak at day 14 (*p* ≤ 0.001) followed by a decrease at days 21 and 28 (*p* ≤ 0.001). A similar pattern was found in IL-5 CD4^+^ T cells, except at day 28 when levels were no different from mock. IL-6 producing cells in PP showed that bLf treated mice had an initial peak at day 7 (*p* ≤ 0.001), followed by a decrease at days 14, 21 and 28 compared to mock (*p* ≤ 0.05, *p* ≤ 0.001, and *p* ≤ 0.001, respectively). IL-10^+^ CD4^+^ T cells percentages were decreased at days 14 and 28 (*p* ≤ 0.001) in comparison to mock. TGF-β^+^ CD4^+^ T cells percentage was increased at every time point following intervention in comparison to mock (*p* ≤ 0.001). In a non-linear regression model, an overall reduction of Th1 response in the PP was identified.

**Figure 4:**
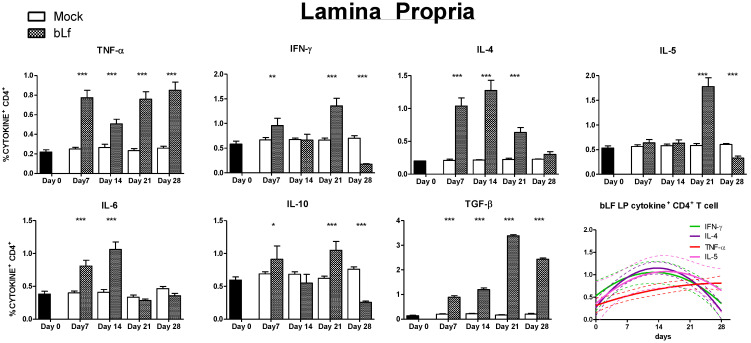
Effects of oral bLf vs. mock on the percentages of cytokine+ CD4+ T cells in LP of the proximal small intestine. Mean and SD are shown. Non-linear regression models were plotted for TNF-α, IFN- γ, IL-4 and IL-5. Significant differences: * *p* ≤ 0.05, ** *p* ≤ 0.01, *** *p* ≤ 0.001 compared to mock. Non-significant results are omitted.

In the effector compartment (LP) of proximal small intestine (Figure 4), TNF-α^+^ CD4^+^ T cells percentages were higher at every timepoint after intervention in comparison to mock (*p* ≤ 0.001) (Figure 4). IFN-γ^+^ CD4^+^ T cells populations from bLf treated mice presented peaks at days 7 and 21 (*p* ≤ 0.001), and lower values at day 28 (*p* ≤ 0.001) in comparison to mock. IL-4 percentages were increased in bLf treated mice at days 7, 14 and 21 (*p* ≤ 0.001) in comparison to mock, showing no difference at day 28. IL-5^+^ CD4^+^ T cells percentages were higher in the bLf group in comparison to mock at day 21 (*p* ≤ 0.001), followed by a marked decrease at day 28 (*p* ≤ 0.001). IL-6^+^ CD4^+^ populations were increased in the bLf treated group at days 7 and 14 (*p* ≤ 0.001) followed by a return to mock levels. IL-10^+^ CD4^+^ T cells percentages showed peaks at days 7 and 21 (*p* ≤ 0.05 and *p* ≤ 0.001, respectively) with a final decrease in comparison to mock at day 28. Finally, TGF-β^+^ CD4^+^ T cells percentages from mice treated with bLf showed an increase in every time point after intervention in comparison to mock-treated mice (*p* ≤ 0.001). In a non-linear regression model, an overall Th2 pattern of augmentation followed by decrease was identified in the proximal small intestine LP of bLf-treated mice. Taken together, analysis of CD4^+^ T cell population shows a different effect in the inductor and effector compartments, as well as across time.

## 4. Discussion

bLf has been proposed as a nutrient intervention with immunomodulatory properties that may be beneficial in immune-mediated disorders of the gut [25,26]. On one side, extensive characterization of its anti-infective properties has been conducted and reviewed [27], while the impact of bLf administration in an otherwise healthy gut has not been widely explored. Importantly, despite the complex dynamics of immunomodulation in the different anatomical compartments of the gut, available data from the murine model of oral administration had been limited to single measurements of immune responses across time and/or short bLf administration courses. Due to this, and in light of the immunological regionalization of the gut mucosa, the current study focused on the murine proximal small intestine, equivalent to the human duodenum and jejunum, a site associated to nutrient absorption with an important role in the induction of mucosal responses.

As expected, oral bLf increased the levels of total IgA and IgM, as well as bLf-specific antibodies. This is in line with previous reports that describe whole intestine secretions [6] as well as isolated distal small intestine [8] from mice that received oral bLf treatment. Remarkably, we found that after initial increase, total IgM levels were no different to mock at day 28. This observation was previously described in the distal small intestine [8] suggesting a regulation of IgM secretion only detected after extended oral administration. In the mentioned study, total IgA in the distal small gut followed a similar pattern as presented here in the proximal section. Interestingly, the same pattern of an initial IgM peak followed by a decreasing trend toward control levels was reported in total intestinal secretions [9], although oral bLf was administered only until day 21. This finding emphasizes the importance of extended monitoring of both specific and nonspecific responses in the gut after bLf administration.

Analysis of the PC population in the LP, the most important effector compartment of the gut mucosa [28], revealed that, despite an initial increase in comparison to mock, both IgA^+^ and IgM^+^ PC were below control levels at day 28. While antibody production is not limited to the LP and other sites may be responsible for total IgA and IgM production [23], differences across time points suggest that a dynamic control leading to downregulation of survival/differentiation signals at the proximal small gut may be present. The presence of a low frequency population of PC with intensive IgA-producing properties or extended life span may be also a possibility [29]. As such specialization would require important changes in the cytokine microenvironment that favors a robust humoral response, we analyzed the percentage of CD4^+^ T cells on the effector compartment, in contraposition to inductor sites and cytotoxic T cell populations. CD4^+^ T cells percentages in the LP were increased in comparison to the CD8^+^ T cells and mock, indirectly supporting such hypothesis; therefore, we decided to explore the cytokine producing populations in both LP and PP, as the latter have a shaping role in the direction and strength of the immune response in the effector sites [30].

We divided the inductor and effector site analysis of the proximal small intestine in Th1 and Th2 associated response, an inflammatory related response (IL-10 and IL-6), and TGF-β, as a master gut immunoregulatory cytokine [31]. Concerning Th1 response, we found that there is a general downregulation in the inductor site. In the effector sites, bLf influence on Th1 responses is less straightforward, as waves of cytokine producing CD4^+^CD3^+^ T cells population appear to be present. This same pattern was described previously on similar bLf intervention on the distal small intestine [8] and may indicate an immunomodulatory tuning between stimulation and inhibition. Such dynamic response may limit Th1 related immunopathogenesis as well as counteracting excessive Th2 reactions as it was reported in an allergy model [32,33].

With regard to Th2 response associated cytokines, we detected an initial surge in the inductor compartment. Even more importantly, both IL-4 and IL-5, critical enhancers of IgA production in the gut, repeated the same surge-reduction pattern in the effector compartment, although at different time points. Unexpectedly, the reduction of the Th2 cytokine-producing CD4^+^ T cell population in the LP at day 28 matched the time point where specific mucosal humoral responses against bLf were highest. Other cell populations could be the source of these important Th2 cytokines that support antibody production in the small intestine. Innate lymphoid cells (ILC) may have a role [34] and it is possible that bLf exerts some effects due to its pleiotropic abilities. The complex outline of Th1/Th2 response in the effector sites, points out the necessity of including time-dependent immunomonitoring as incomplete readout may obscure the interpretation of the effects of oral bLf. This may explain the apparent conflicting results that describe oral bLf as Th1 or Th2 enhancer or repressor [6,8,11,13,14,35,36]. Importantly, the effect may not only depend on the immune context (inflammation vs. non-inflammation [32]) but also on the anatomical site, as we find clear differences in cytokine CD4^+^ T cell population between the proximal gut patterns described in this study, and distal small gut findings previously reported by our group [8].

Oral bLf regulatory effect on noninfectious inflammatory intestinal conditions have been extensively explored [14,37,38,39,40,41]. IL-10 is the archetypal anti-inflammatory cytokine in the gut [42] and its production by CD4^+^ T cells has been proposed as a downregulatory “master” molecule [43]. We found that in the proximal small intestine of bLf-treated mice, IL-10^+^ CD4^+^ T cell populations are decreased at day 28 in comparison to mock. Surprisingly, we found that in the effector site two peaks were present at days 7 and 21. A positive effect of a 7-day oral bLf course on IL-10 production of intestinal intraepithelial lymphocytes and mesenteric lymph node lymphocytes was previously described [11], but no insight on longer administration was reported. On the other hand, we found a transitory elevation of the population of IL-6 producing CD4^+^ T cells in the effector site. Remarkably, it has been suggested that CD4^+^ T cells IL-6 production may be associated to a Th2 response [44] so its significance as an inflammatory marker may be oversimplified.

Finally, we decided to look into in the TGF-β CD4^+^ T cell population, as this cytokine has Th17 and Treg-inducing properties as well as powerful effects on the control of IgA production [31]. We found that both inductor and effector sites of the proximal small intestine from bLf treated mice had higher percentages of TGF-β^+^ cells in comparison to mock. Although bLf has been shown to interact with the TGF-β receptor and activate canonical TGF-β signaling [45,46,47], this is to our knowledge the first evidence of upregulation of TGF-β production in immune cells associated to oral bLf in the healthy gut mucosa. This observation may help to explain the divergent effect of bLf on bLf-specific and total IgA and the reduction of IgA^+^ PC at the end of the intervention. It is possible that via direct bLf interaction or through TGF-β production stimulation, an increase in the frequency of IgA-secreting B-cell clones takes place, while inhibition of unspecific B-cell mitogenic pathways derived from ubiquitous signals as LPS or CpG occurs [46]. More importantly, TGF- β influence in the gut extends beyond lymphoid cell regulation, including predominant roles in the maintenance of epithelial homeostasis, gut-microbiota adequate interaction and intestinal barrier preservation. This may point to a possible beneficial effect on gut pathologies such as inflammatory bowel disease and colitis-associated colon cancer, where TGF-β dysfunction has been found [31]. Although an enticing possibility, this initial observation was done in an otherwise healthy mouse, and requires further investigation including analysis of multiple cell populations and mucosal sites, as well as adequate models that resemble the inflammatory characteristics. Recent work conducted in that direction confirms that bLf has a beneficial effect in such conditions and that TGF-β may have a role [41,48].

By analyzing cytokine producing CD4^+^ T cells during extended oral bLf administration, we reveal a complex pattern of surges and reductions in different populations of both effector and inductor compartments of the proximal small gut. As CD4^+^ T cells migration, survival, and proliferation depends on the gut microenvironment, manifested in the cytokine milieu [33], characterization of this population allows a functional overview of the effect of bLf on proximal small intestine immunity. Therefore, changes on the cytokine^+^ CD4^+^ T cells act as an overall marker of the pleiotropic effects of bLf on the gut. CD4^+^ plasticity, via cytokine-driven reprogramming, supports this model in which bLf-modified microenvironment modulates gut immunity dynamically. bLf dissimilar and even contrasting impact in inductor and effector sites indicates that this outcome is conditional to immunological site and intestinal region.

We are aware of the limitation of the isolated analysis of CD4^+^ T cells, exemplified by the heterogeneity of IL-10 producing CD4^+^ T cells that may encompass both anti-inflammatory and proinflammatory populations [49]. Moreover, the paradigmatic model of Th1/Th2 response, even after the inclusion of Th17, Th9, Th22, Tfh, Treg and Tr1 populations [24] has been put into question and recent evidence pointing the continuum phenotype of gut CD4^+^ T cells [50] shows the shortcomings of this model. It is important to mention that the division of the gut into effector and inductor compartments is also artificial as the lamina propria can also act as an inductor site [51]. Nevertheless, the Th model had shown relevance in determining a particular signature of immune diseases, as well as directing therapeutic interventions and monitoring. Although our findings are based on a healthy murine gut, and Th signature of oral bLf may radically change in disease models, this approach better resembles the scenario where bLf is a nutrient in healthy subjects, as opposed to therapeutic interventions.

Considering that extended oral bLf administration induces immune changes in the proximal gut, and that the European Commission has approved bLf as a novel food ingredient [52], future studies may need to use even longer intervention times to better resemble the long-term impact of bLf on mucosal immunity. As bLf effect may be dependent on both the intact molecule and its digestion-derived peptides, bLf enteral administration and its interaction with diet should also be examined to determine how this may affect bLf immumodulatory properties.

## 5. Conclusions

To conclude, we used an oral bLf administration murine model to explore its immunological properties on the healthy proximal small intestine across several time points in a 28-day period of intervention. We found evidence of dynamic changes in the cytokine-secreting CD4^+^ T cell population that indicates the role of bLf as a natural immunomodulatory nutritional intervention. Whether such effects are beneficial in the long term, have to be independently tested on different models of mucosal diseases, taking into account the temporal dynamic changes in the different gut compartments and regions. We provide proof of the relevance of mucosal immunomonitoring of nutraceutical interventions as a requirement for improved understanding of the consequences of their administration.

## Data Availability

The data presented in this study are available on request from the corresponding author.

## Supplementary Materials

The following are available online at https://www.mdpi.com/article/10.3390/nu13082852/s1, Figure S1: Gating strategy for analysis of CD4^+^ and CD8^+^ T cells; Figure S2: Gating strategy for intracellular cytokines of CD4^+^ T cells in lamina propria of the proximal small intestine.
