# Supplementary material for: Temporal Dynamics of T Helper Populations in the Proximal Small Intestine after Oral Bovine Lactoferrin Administration in BALB/c Mice
Source: Nutrients. 2021 Aug 19;13(8):2852. doi: 10.3390/nu13082852 (PMC8399302; doi:10.3390/nu13082852)
Supplement: Supplementary file 1 [file nutrients-13-02852-s001.zip › nutrients-1308822-supplementary.pdf]

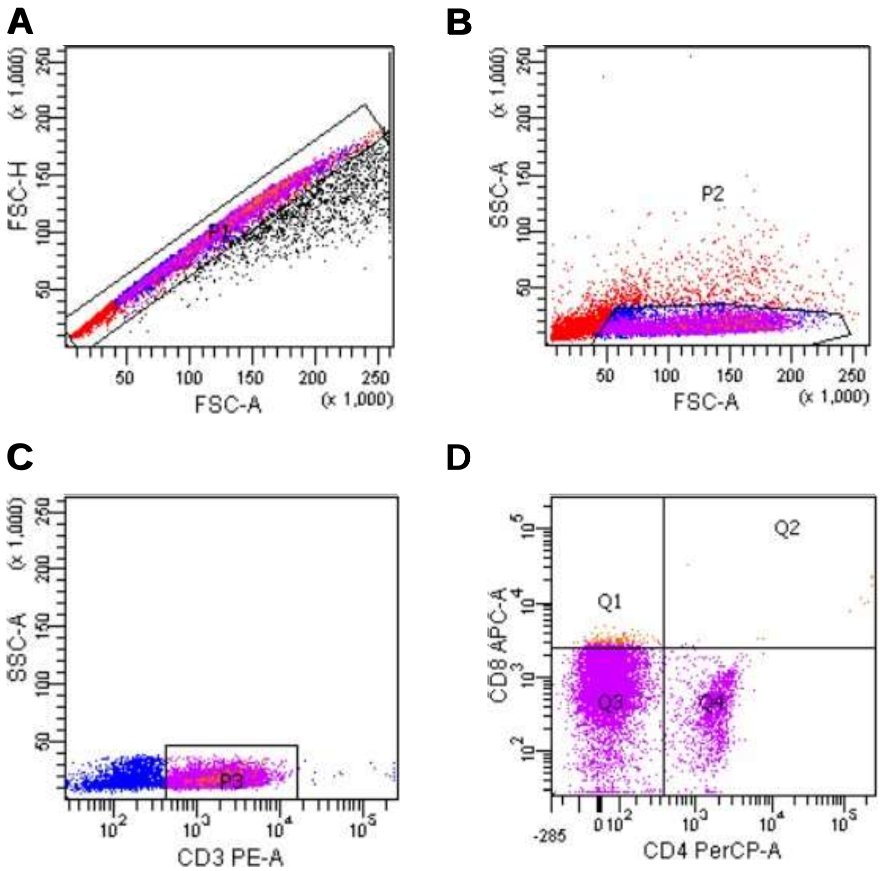

Figure S1. Gating strategy for analysis of CD4+ and CD8+ T cells. Single cells were gated using FSC-A and FSC-H (A). Cell were gated based on size and granularity using FSC-A and SSC-A to determinate lymphocyte region (B). CD3+ (PE) T cells were selected for further analysis (C). Subsequently, CD4+ (PerCP) and CD8 (APC) + T cell was analyzed.

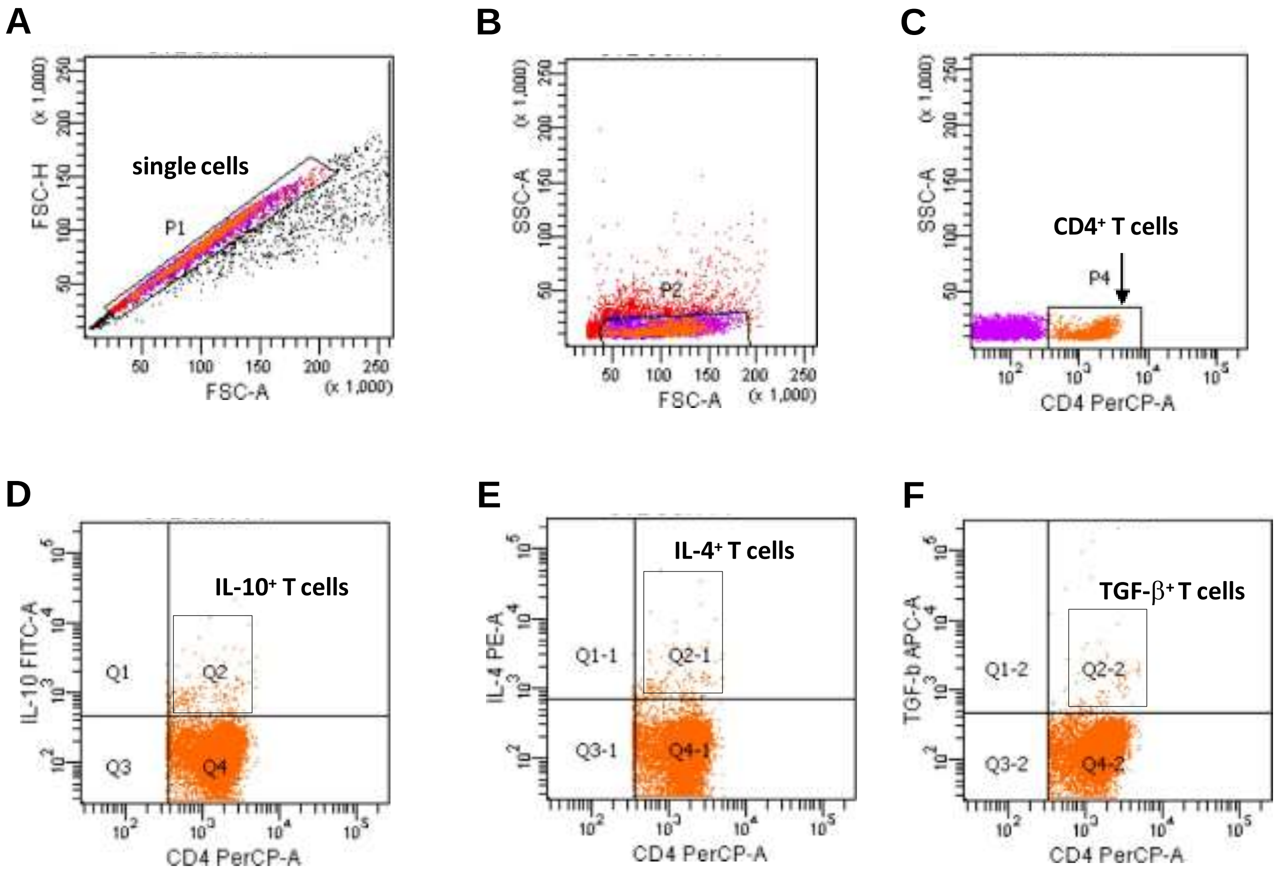

Figure S2. Gating strategy for intracellular cytokines of CD4+ T cells in lamina propria of the proximal small intestine. Single cells were gated using FSC-A and FSC-H (A). Cell were gated based on size and granularity using FSC-A and SSC-A to determinate lymphocyte region (B). CD4+ (PerCP) T cells were selected for further analysis (C). Subsequently, CD4+ (PerCP) T cells was followed by examination of expression of IL-10 (FITC) (D), IL-4 (PE) (E) and TGF-β (APC) (F)
